# Supplementary material for: Genomic insights into recent species divergence in Nicotiana benthamiana and natural variation in Rdr1 gene controlling viral susceptibility
Source: Plant J. 2022 May 31;111(1):7–18. doi: 10.1111/tpj.15801 (PMC9543217; doi:10.1111/tpj.15801)
Supplement: Supplementary file 1 — Figure S1. RAxML tree of N. benthamiana. [file TPJ-111-7-s003.pdf]

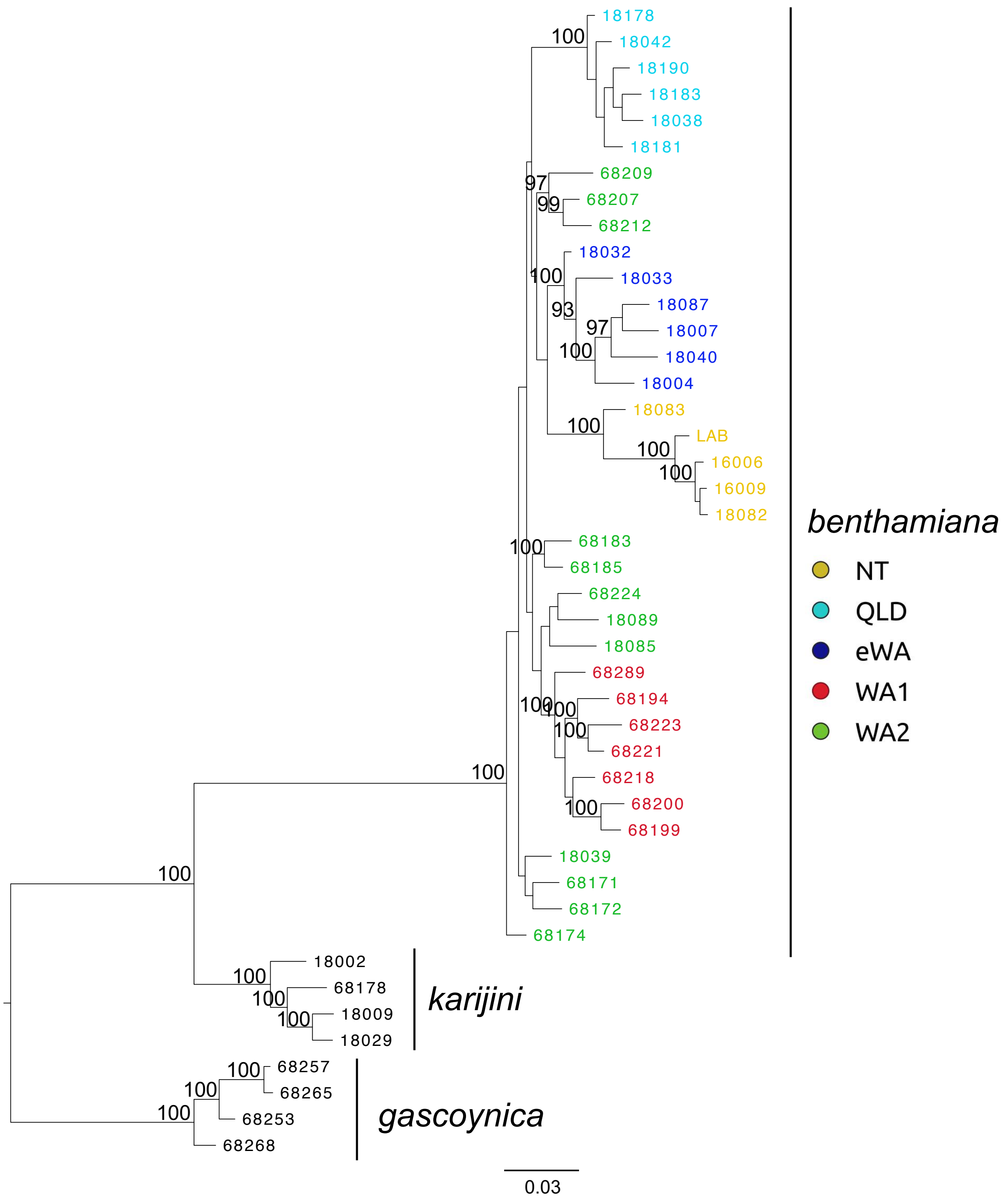

Supplementary Fig. 1. RAxML tree based on 14,285 SNPs. The colour of the clusters represents their geographical provenances: yellow – Northern Territory and northeastern-most Western Australia (NT); dark blue – the deserts of eastern Western Australia (eWA); light blue – Queensland and western-most northern Territory (QLD); red - Pilbara coast of northwestern Western Australia (WA1) and; green - the Pilbara Craton of Western Australia

(WA2), the last unresolved but the interrelations of these accessions and their relationships to the other groups are not well supported.
