# Supplementary material for: Genomic insights into recent species divergence in Nicotiana benthamiana and natural variation in Rdr1 gene controlling viral susceptibility
Source: Plant J. 2022 May 31;111(1):7–18. doi: 10.1111/tpj.15801 (PMC9543217; doi:10.1111/tpj.15801)
Supplement: Supplementary file 2 — Figure S2. Delta K values for the best K model obtained in NGSadmix. [file TPJ-111-7-s005.pdf]

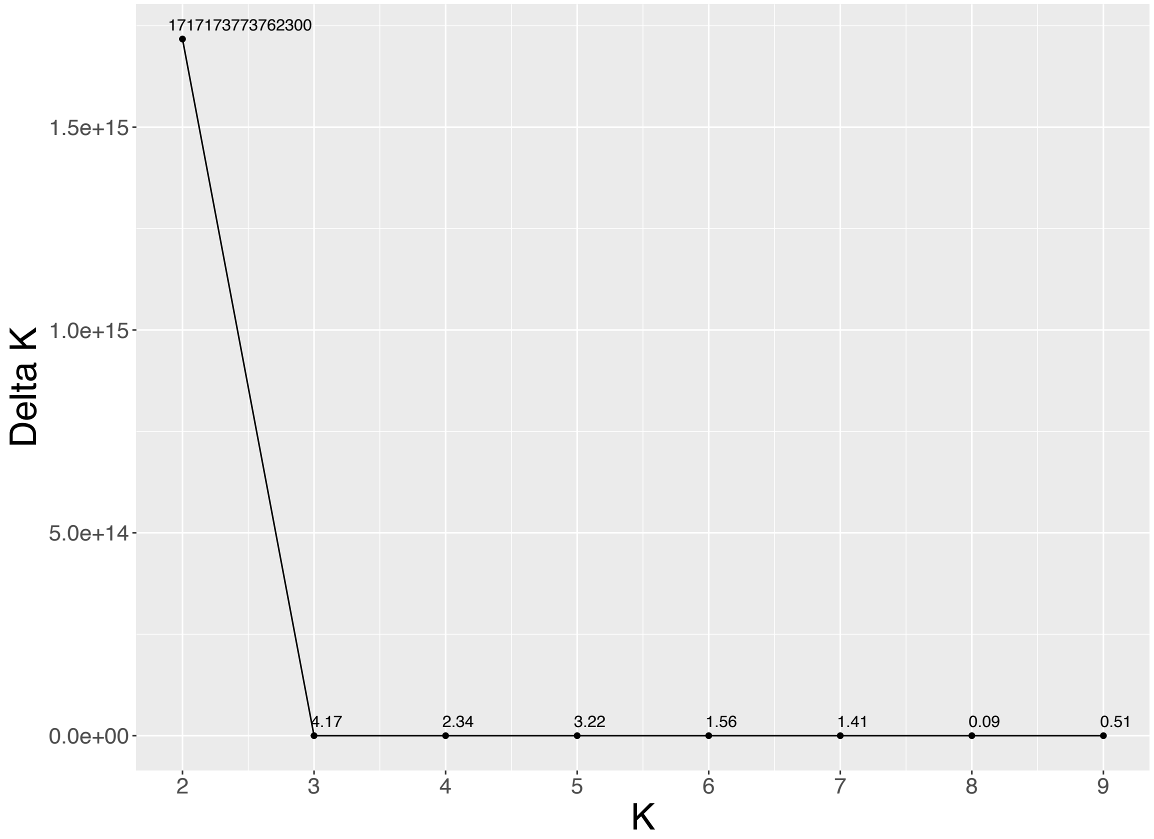

Supplementary Fig. 2. Delta K values (y-axis) in respect to K values (x-axis) obtained in NGSadmix. The best K was estimated in accordance with the Evanno method (http://clumpak.tau.ac.il/bestK.html).
