# Supplementary material for: Genomic insights into recent species divergence in Nicotiana benthamiana and natural variation in Rdr1 gene controlling viral susceptibility
Source: Plant J. 2022 May 31;111(1):7–18. doi: 10.1111/tpj.15801 (PMC9543217; doi:10.1111/tpj.15801)
Supplement: Supplementary file 3 — Figure S3. Structure plots of N. benthamiana groups obtained in NGSadmix. [file TPJ-111-7-s006.pdf]

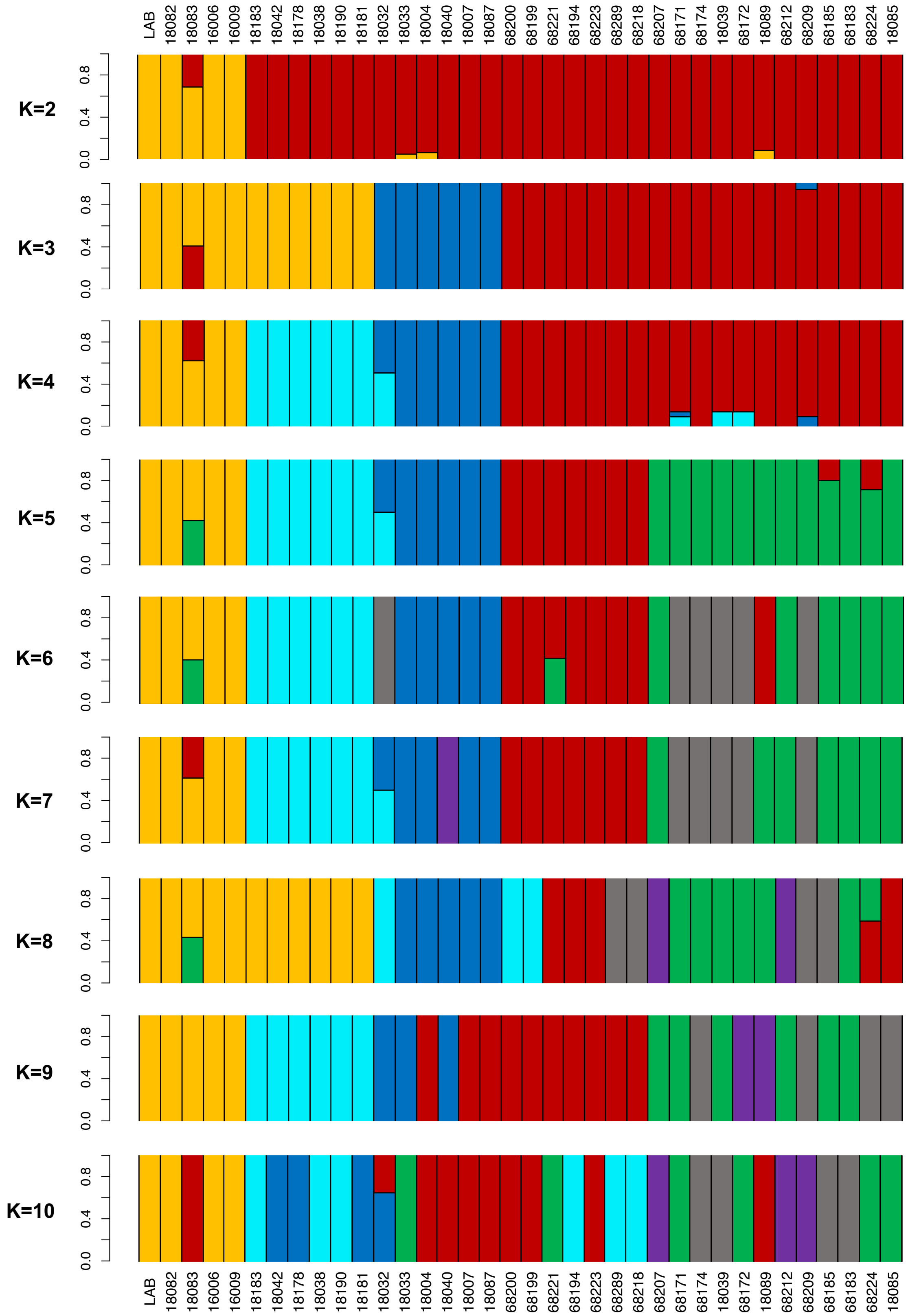

Supplementary Fig. 3. Structure plots of *N. benthamiana* groups obtained in NGSadmix for K = 2 through 10. The values on y-axis represent admixture proportions.
