# Supplementary material for: Genomic insights into recent species divergence in Nicotiana benthamiana and natural variation in Rdr1 gene controlling viral susceptibility
Source: Plant J. 2022 May 31;111(1):7–18. doi: 10.1111/tpj.15801 (PMC9543217; doi:10.1111/tpj.15801)
Supplement: Supplementary file 4 — Figure S4. Coancestry heatmap of N. benthamiana and the most closely related species N. gascoynica and N. karijini. [file TPJ-111-7-s008.pdf]

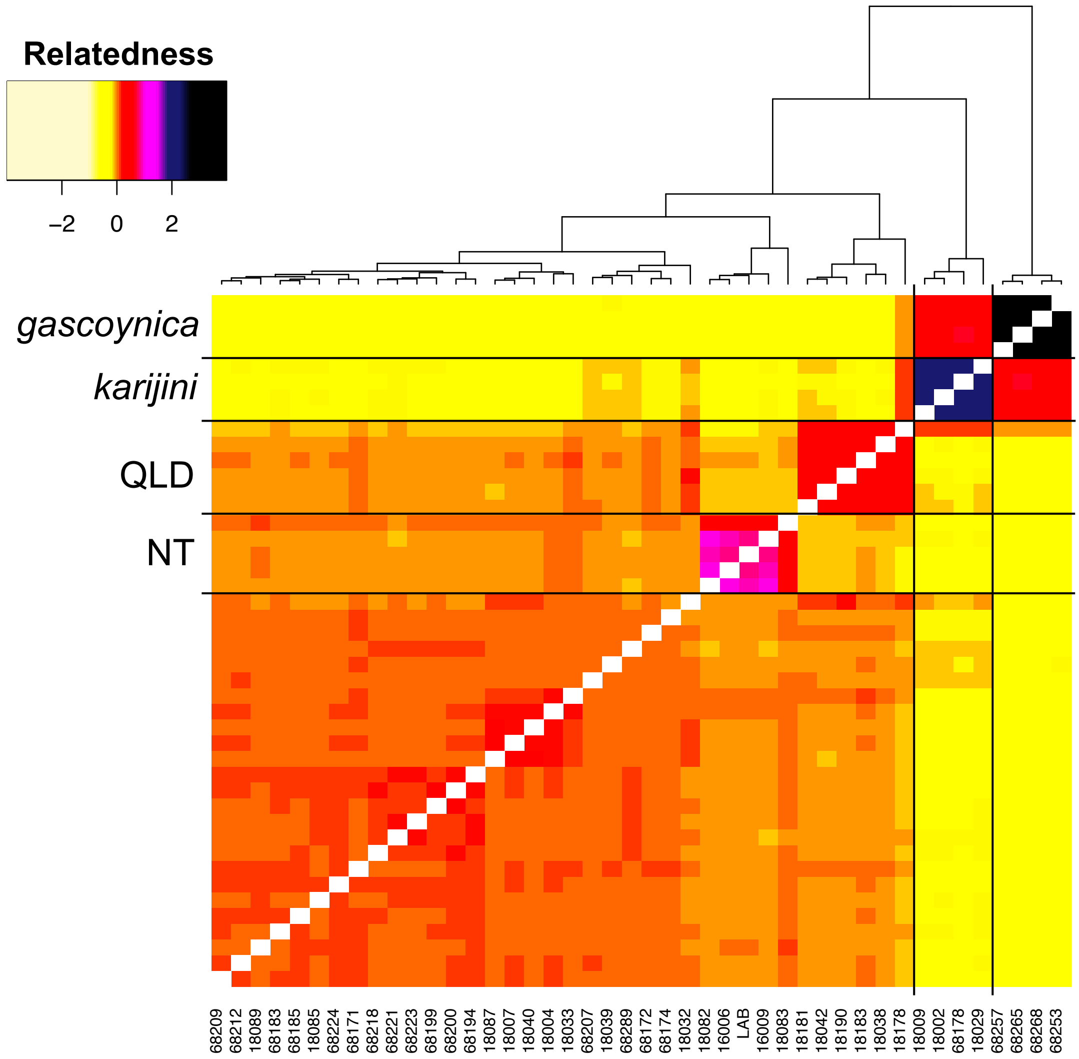

Supplementary Fig. 4. Coancestry heatmap of *N. benthamiana* and the closest related species *N. gascoynica* and *N. karijini*. The heatmap was constructed based on genotype likelihoods obtained in ANGSD. Darker tones represent higher pairwise relatedness according to legend; estimates for the relationship of one individual to itself have been excluded.
