# Supplementary material for: Genomic insights into recent species divergence in Nicotiana benthamiana and natural variation in Rdr1 gene controlling viral susceptibility
Source: Plant J. 2022 May 31;111(1):7–18. doi: 10.1111/tpj.15801 (PMC9543217; doi:10.1111/tpj.15801)
Supplement: Supplementary file 5 — Figure S5. Maximum likelihood tree produced by TreeMix showing a migration event (arrow) between N. karijini and the QLD accessions. [file TPJ-111-7-s002.pdf]

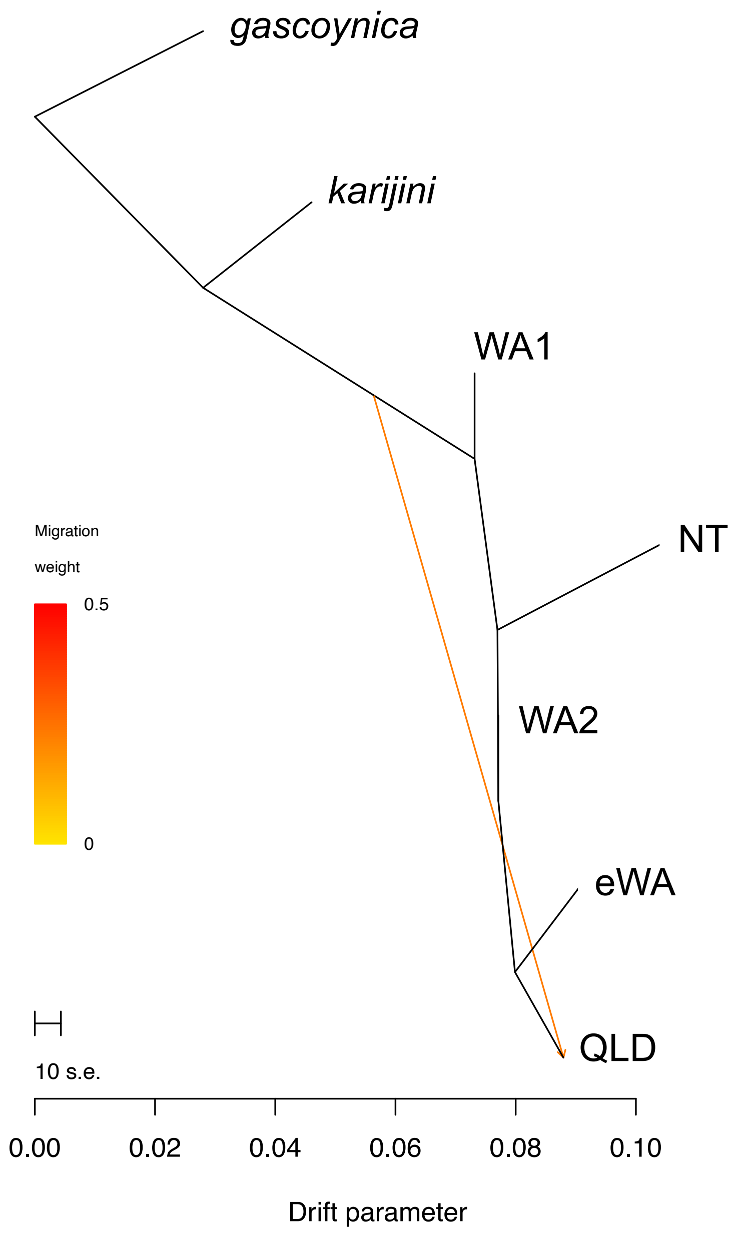

Supplementary Fig. 5. Maximum-likelihood tree produced by TreeMix showing a migration event (arrow) between *N. karijini* and the group *N. benthamiana* of accessions from Queensland (QLD). The weight of migration edges is shown in the colour scale, with darker tones representing greater estimates of migration. The drift parameter corresponds to the amount of genetic drift that has occurred along each branch.
