# Supplementary material for: Genomic insights into recent species divergence in Nicotiana benthamiana and natural variation in Rdr1 gene controlling viral susceptibility
Source: Plant J. 2022 May 31;111(1):7–18. doi: 10.1111/tpj.15801 (PMC9543217; doi:10.1111/tpj.15801)
Supplement: Supplementary file 6 — Figure S6. Models and accessions used in the Bayesian species delimitation analysis in SNAPP. [file TPJ-111-7-s007.pdf]

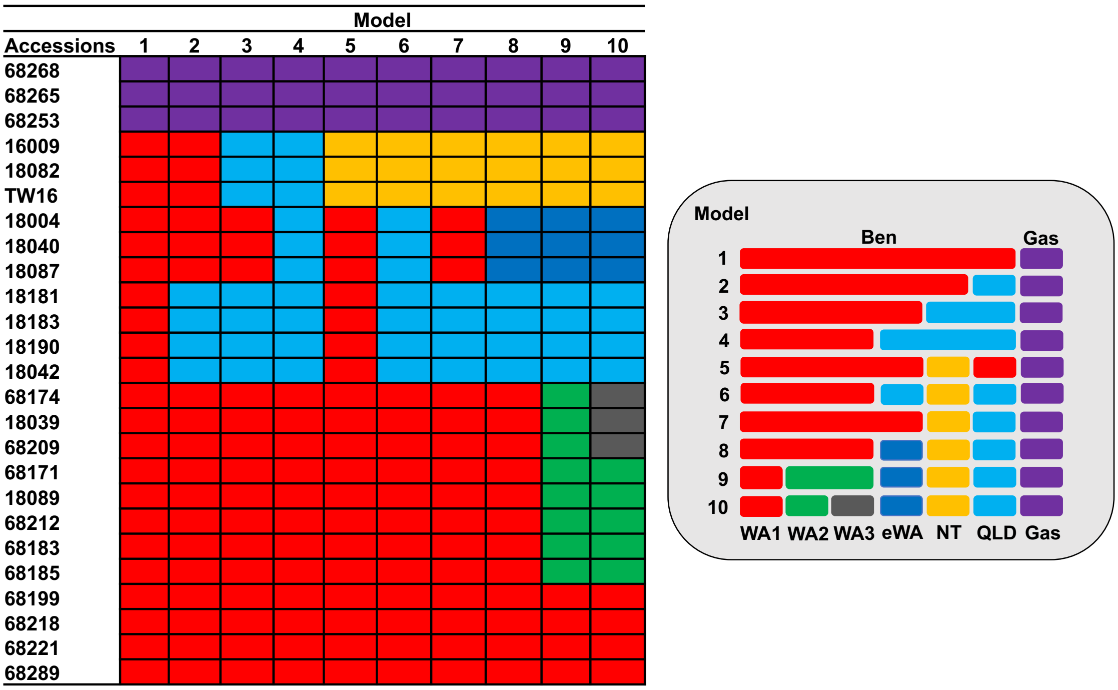

Supplementary Fig. 6. Schematic representation of models and accessions assigned to each particular species group in the Bayesian species delimitation performed in SNAPP. The split of *N. benthamiana* in potential new species were tested in ten models and the evaluated new species were represented with different colors. The greatest split comprises six species in the *N. benthamiana* complex (model 10) represented by accessions from: yellow – Northern Territory and north-eastern-most Western Australia (NT); dark blue – the deserts of eastern Western Australia (eWA); light blue – Queensland and western-most northern Territory (QLD); red - Pilbara coast of northwestern Western Australia (WA1); green and grey- two populations from the Pilbara Craton of Western Australia (WA2 and WA3). *Nicotiana gascoynica* (GAS) is a related, distinct species (Supplementary Figure 2).
