# Supplementary material for: Genomic insights into recent species divergence in Nicotiana benthamiana and natural variation in Rdr1 gene controlling viral susceptibility
Source: Plant J. 2022 May 31;111(1):7–18. doi: 10.1111/tpj.15801 (PMC9543217; doi:10.1111/tpj.15801)
Supplement: Supplementary file 7 — Table S1. Voucher numbers, herbaria and provenance of the plant material. [file TPJ-111-7-s004.docx]

Table S1. Voucher numbers, herbaria and provenance of the plant material. New species names accord with those proposed in Chase et al. (submitted)

| **Species name** | **Voucher number (*Chase & Christenhus*)*** | **Latitude/longitude (S and E; degrees, minutes, seconds)** | **Provenance (brief locality name, all Australia)** |
| --- | --- | --- | --- |
| *benthamiana* | *16006* | -15, 36, 22; 131, 5, 3 | Judbarra Gregory NP, Northern Territory |
| *benthamiana* | *16009* | -15, 36, 45; 131, 8, 57 | Judbarra Gregory NP, Northern Territory |
| *rupestris* | 18004  *Butcher & Albrecht 2048* (PERTH 8855234) | -22, 44, 39; 126, 36, 27 | 115.3 to 118.5 km due West of Kiwirrkurra, Gary Junction Road, Western Australia |
| *rupestris* | *18007*  *Muir 1023* (PERTH 8610134) | -24, 4, 9.75; 123, 9, 58.47 | 400 k E of Capricorn, Moongooloo Rock Hole, Constance Headland, Western Australia |
| *rupestris* | *18032*  *Latz 30766* (NT D0273011); | -24, 24, 18; 127, 45, 58 | Circus Rockhole, Rawlinson Range, Western Australia |
| *rupestris* | *18033*  *Goods 1145* (PERTH 8894183), | -22, 0, 57.3; 127, 44, 38.8 | 4 km S of Bibarrd, Western Australia |
| *scopulorum* | *18038*  *Albrecht 12441* (NT D0182096) | -23, 3, 20; 136, 58, 58 | Mt. Tietkens, North Simpson Desert, Northern Territory |
| *bilybara* | *18039*  *Bean 25412* (BRI AQ735848; PERTH 8116067 | -22, 52, 55; 119, 14, 9 | E of Weeli Wolli Creek, 75 km NW of Newman, Western Australia |
| *rupestris* | *18040*  *Latz 22902* (NT D0182743) | -24, 58, 0; 129, 8, 52 | 12 km SE of Docker River Settlement, Learmonth Park, Northern Territory |
| *scopulorum* | *18042*  *Wannan 5860* (BRI AQ0855406) | -16, 40, 43; 144, 12, 29 | Beside tributary of Elizabeth Ck, Bellevue, Queensland |
| *benthamiana* | *18082*  *Cowie 13343* (CANB 595117.1) | -14, 18, 41; 130, 58, 17 | Fish River Station, Northern Territory |
| *benthamiana* | *18083*  *Willing s.n.* (PERTH 8557993) | -14, 2, 12; 127, 19, 31 | King George River tidal estuary, N Kimberley, Western Australia |
| *bilybara* | *18085*  *McMaster 25736* (PERTH 8608652) | -22, 11, 35; 116, 14, 14 | Site API-5011. Cardo East, West Pilbara Iron project area, Western Australia |
| *rupestris* | *18087*  *Davis 11189* (CANB 698209.1) | -23, 45, 16; 122, 30, 51 | Durba Springs, Canning Stock Route, Western Australia |
| *scopulorum* | *18178* | -20, 53, 7; 140, 20, 59 | Duchess/Cloncurry Road, SSW of Cloncurry, Queensland |
| *scopulorum* | *18181* | -21, 23, 8; 139, 49, 53 | Duchess/Dajarra Road, 5 km southwest of Duchess, Queensland |
| *scopulorum* | *18183* | -21, 6, 47; 139, 48, 54 | Duchess/Mount Isa Road, 55 km southeast of Mount Isa, Queensland |
| *scopulorum* | *18190* | -20, 35, 12; 139, 34, 41 | Lake Moondara Park, Queensland |
| *bilybara* | *6817*1 | -23, 17, 3; 119, 39, 30 | Silent Gorge, ca 10 km west of Newman, Western Australia |
| *bilybara* | *68172* | -23, 9, 5; 119, 20, 5 | Great Northern Highway (95) 11 km from Newman, Western Australia |
| *bilybara* | *68174* | -23, 2, 27; 118, 51, 4 | Mt Robinson, Western Australia |
| *bilybara* | *68183* | 22, 21, 30; 118, 17, 4 | Karijini National Park, Hancock Gorge, Western Australia |
| *bilybara* | *68185* | -22, 21, 25; 118, 17, 13 | Karijini National Park, Weano Gorge, Western Australia |
| *candelabra* | *68194* | -21, 34, 11; 117, 3, 8 | Millstream-Chichester National Park, Western Australia |
| *candelabra* | *68199* | -20, 50, 22; 117, 8, 13 | Road from Roebourne to Harding Dam, Western Australia |
| *candelabra* | *68200* | -20, 53, 19; 117, 20, 26 | ca 2 km WNW of Wittenoom turn off, NW Coastal Highway, Western Australia |
| *bilybara* | *68207* | -21, 21, 42; 118, 42, 55 | Great Northern Highway, Western Australia |
| *bilybara* | *68209* | -21, 33, 44; 119, 19, 14 | Woodstock-Marble Bar road, Western Australia |
| *bilybara* | *68212* | -21, 19, 54; 119, 35, 28 | Woodstock-Marble Bar road, Western Australia |
| *candelabra* | *68218* | -20, 50, 16; 117, 53, 21 | NW Coastal Hwy, ca. 5 K E of Whim Creek, Western Australia |
| *candelabra* | *68221* | 21, 3, 12; 116, 15, 12 | Fortescue River Mouth Road, Western Australia |
| *bilybara* | *68223* | -21, 38, 4; 116, 0, 35 | Pannawonica Road, Western Australia |
| *bilybara* | *68224* | -21, 39, 39; 116, 16, 30 | Pannawonica Road, west of Pannawonica, Western Australia |
| *candelabra* | *68289* | -21, 20, 2; 117, 17, 18 | Millstream-Chichester National Park, Western Australia |
| *bilybara* | *18089*  *Chinnock 9599* (AD 170035; NT A0109792; PERTH 7094582) | -21, 12, 13; 121, 1, 21 | Oakover River on Woodie Woodie Rd, Western Australia |
| *benthamiana* LAB | TW16, vouchered at K, *18139* | -20, 34, 14; 130, 21, 13† | United States Department of Agriculture seed bank |
| *gascoynica* | *68253* | -24, 49, 41; 113, 46, 12 | NW Coastal Hwy, Gascoigne River Crossing, Western Australia |
| *gascoynica* | *68257* | -24, 45, 21; 114, 8, 10 | Gascoyne River, Rocky Pool, Western Australia |
| *gascoynica* | *68265* | -25, 17, 46; 115, 36, 38 | Carnarvon-Mullewa Road, Daurie River crossing, Western Australia |
| *gascoynica* | *68268* | -25, 45, 29; 114, 16, 41 | NW Coastal Hwy, Wooramel River Bridge, Western Australia |
| *karijini* | *18002*  *Naaykens 15-5-J280* (PERTH 8757682) | 23, 15, 56; 117, 44, 18.7 | Eastern Range/Channar, Greater Paraburdoo, Pilbara, Western Australia |
| *karijini* | *18009*  *Anderson 172* (PERTH 8437386) | 22, 42, 4.7; 117, 23, 58.59 | 1.6 km NE of Mount Turner, Western Australia |
| *karijini* | *18029*  *Fairhead & Venkatasamy 0071* (PERTH 8430861) | 23, 12, 0; 117, 30, 0 | 21.7 km W of Paraburdoo, Western Australia |
| *karijini* | *68178* | 22, 23, 25; 118, 16, 3 | Karijini National Park, Joffre Gorge, Western Australia |

*unless otherwise indicated; vouchers deposited at PERTH, DNA/NT or BRIS, depending on the state in which they were collected. †Coordinates for the Granites Goldmine, Northern Territory. Accessions raised from seeds retrieved from herbarium material are documented by secondary vouchers at PERTH, DNA/NT, BRIS or Royal Botanic Gardens, Kew (K); for these, the original collector, number and herbarium accession number in Australia are also provided.
