# Supplementary material for: Genomic insights into recent species divergence in Nicotiana benthamiana and natural variation in Rdr1 gene controlling viral susceptibility
Source: Plant J. 2022 May 31;111(1):7–18. doi: 10.1111/tpj.15801 (PMC9543217; doi:10.1111/tpj.15801)
Supplement: Supplementary file 8 — Table S2. Rdr1 presence/absence, seed sizes and germination, flowering and capsule maturation times for a selection of relevant accessions and species of Nicotiana sect. Suaveolentes. [file TPJ-111-7-s001.docx]

Table S2. *Rdr1* presence/absence, seed sizes and germination, flowering and capsule maturation times for a selection of relevant accessions and species of *N.* sect. *Suaveolentes*. These data were collected for all 36 accessions of the *N. benthamiana* species group studied here, but we do not include all these due to their highly redundant nature. Note: *Nicotiana simulans* and *N. exigua* are distantly related to the *N. benthamiana* complex and do not carry the *Rdr1* insertion, but they develop and mature much more quickly compared to the species of the *N. benthamiana* complex.

| **Accession** | **Group** | ***Rdr1* insertion** | **Seed size (microns)** | **Days to germination** | **Days to flowering** | **Days to capsule maturation** |
| --- | --- | --- | --- | --- | --- | --- |
| LAB | NT | present | 790–805 | 3 | 55 | 87 |
| *Chase & Christenhusz 16006* | NT | absent | 650–680 | 4 | 53 | 87 |
| *Chase & Christenhusz 16009* | NT | present | 715–755 | 4 | 54 | >87 |
| *Cowie 13343* (18082)* | NT | present | 720-780 | 5 | 54 | >90 |
| *Chase & Christenhusz 18190** | QLD | absent | 530–590 | 4 | 62 | >90 |
| *Chase & Christenhusz 18183** | QLD | absent | 530–585 | 4 | 58 | >90 |
| *Latz 22902 (18040)* | eWA | absent | 510–565 | 5 | 50 | >87 |
| *Goods 1145* (18033)* | eWA | absent | 505–555 | 5 | 64 | >90 |
| *Bean 25412 (18039)* | WA2 | absent | 520–585 | 5 | 60 | >90 |
| *Chase & Christenhusz 68174* | WA2 | absent | 520–580 | 5 | 60 | >90 |
| *Chase & Christenhusz 68199* | WA1 | absent | 530–580 | 5 | 58 | >90 |
| *McMaster 25736 (18085)* | WA1 | absent | 535–585 | 6 | 58 | >90 |
| *N. simulans** | n/a | absent | 580–620 | 3 | 35 | 72 |
| *N. exigua** | n/a | absent | 450–485 | 3 | 37 | 75 |

*cleistogamous flowers produced before normal (chasmogamous) flowers.
